# Colorectal Cancer Cell-Derived Small Extracellular Vesicles Educate Human Fibroblasts to Stimulate Migratory Capacity

**DOI:** 10.3389/fcell.2021.696373

**Published:** 2021-07-15

**Authors:** Stefano Piatto Clerici, Maikel Peppelenbosch, Gwenny Fuhler, Sílvio Roberto Consonni, Carmen Veríssima Ferreira-Halder

**Affiliations:** ^1^Department of Biochemistry and Tissue Biology, Institute of Biology, University of Campinas, Campinas, Brazil; ^2^Department of Gastroenterology and Hepatology, Erasmus University Medical Center Rotterdam, Rotterdam, Netherlands

**Keywords:** colorectal cancer, small extracellular vesicles, tumor education, Lmwptp, cancer-associated fibroblast, tumor microenvironment

## Abstract

Colorectal cancer (CRC) is in the top 10 cancers most prevalent worldwide, affecting equally men and women. Current research on tumor-derived extracellular vesicles (EVs) suggests that these small extracellular vesicles (sEVs) play an important role in mediating cell-to-cell communication and thus potentially affecting cancer progression *via* multiple pathways. In the present study, we hypothesized that sEVs derived from different CRC cell lines differ in their ability to reprogram normal human fibroblasts through a process called tumor education. The sEVs derived from CRC cell lines (HT29 and HCT116) were isolated by a combination of ultrafiltration and polymeric precipitation, followed by characterization based on morphology, size, and the presence or absence of EV and non-EV markers. It was observed that the HT29 cells displayed a higher concentration of sEVs compared with HCT116 cells. For the first time, we demonstrated that HT29-derived sEVs were positive for low-molecular-weight protein tyrosine phosphatase (Lmwptp). CRC cell-derived sEVs were uptake by human fibroblasts, stimulating migratory ability *via* Rho-Fak signaling in co-incubated human fibroblasts. Another important finding showed that HT29 cell-derived sEVs are much more efficient in activating human fibroblasts to cancer-associated fibroblasts (CAFs). Indeed, the sEVs produced by the HT29 cells that are less responsive to a cytotoxic agent display higher efficiency in educating normal human fibroblasts by providing them advantages such as activation and migratory ability. In other words, these sEVs have an influence on the CRC microenvironment, in part, due to fibroblasts reprogramming.

## Introduction

Colorectal cancer (CRC) is the third most frequently diagnosed cancer type around the world, resulting in at least 800,000 related deaths occurring in 2018 (Bray et al., 2018). This makes CRC the fourth leading cause of cancer-related deaths in the world (Buccafusca et al., 2019). More than 40,000 new cases of CRC will affect the Brazilian population in 2020/22 (Instituto Nacional de Câncer José Alencar Gomes da Silva (INCA), 2019). CRC patients are expected to have a high life expectancy with an overall 5-year survival rate of 64.9%, but approximately 25% of CRC patients present metastatic events at distant organs resulting in chemoresistance, poor prognostic, and lower survival rate (Buccafusca et al., 2019).

The actual understanding of metastatic events revealed a process that involved many actors and complex cross talk between the cancer cells and their surrounding microenvironment, called tumor microenvironment (TME), which is constituted by a variety of multiple and heterogeneous populations of resident and infiltrating host cells (Hida et al., 2018; Gok Yavuz et al., 2019; Hinshaw and Shevde, 2019; Kobayashi et al., 2019), secreted factors, and extracellular matrix (Eble and Niland, 2019).

In the present study, we focused on two players that can be found in TME, cancer-associated fibroblasts (CAFs), and small extracellular vesicles (sEVs). sEVs are vesicles smaller than 200 nm, as defined according to Minimal Information for Studies of Extracellular Vesicles 2018 (Théry et al., 2019). CAFs are the major component of TME and support pro-tumoral actions related to tumor progression such as tumor growth, angiogenesis, tumor resistance, and immune suppression (Erez et al., 2010; Quail and Joyce, 2013; Borriello et al., 2017; Tao et al., 2017; Gok Yavuz et al., 2019; Kobayashi et al., 2019) and are now considered an attractive target for therapeutic approaches (Liu et al., 2019). sEVs derived from cancer cells appear as early players of microenvironment reprogramming through the transfer of oncogenic signaling pathway mediators from cancer cells to TME and distant cells *via* a process called tumor education (Kahlert and Kalluri, 2013; Milane et al., 2015; Zhao et al., 2016; Richards et al., 2017; Shephard et al., 2017; Zhang et al., 2018; Milman et al., 2019; Stefanius et al., 2021).

The present study highlights that the CRC cell line (HT29) that is less sensitive to death stimuli in comparison with HCT116 cells (Lopes-Costa et al., 2017; Attoub et al., 2018) produces higher amounts of sEVs, which have biological relevance regarding fibroblasts, as proved by an increase of migration and morphological changes. Accordingly, it was observed that Rho and FAK signaling pathways, which are key mediators of cytoskeletal reorganization, were modulated by the coculture of human fibroblasts with CRC cell-derived sEVs. Of great interest, low-molecular-weight protein tyrosine phosphatase (Lmwptp) was found to be part of cargoes of CRC cell-derived sEVs. This observation opens new avenues regarding the molecular mechanism by which this phosphatase gives an advantage to CRC cells.

## Materials and Methods

### Cells and Culture Conditions

Colorectal cancer cells (HCT116 and HT29) were purchased from the Rio de Janeiro Cell Bank (Rio de Janeiro, RJ, Brazil). HCT116 and HT29 cells were cultured in McCoy’s 5A medium containing 100 U/ml penicillin, 100 μg/ml streptomycin, and 10% fetal bovine serum (FBS) at 37°C in a humidified 5% carbon dioxide atmosphere. Human normal skin fibroblasts were kindly donated by Dr. Gustavo Facchini from Kosmoscience Group (Campinas, Brazil) and cultured in Dulbecco’s modified Eagle medium (DMEM) with high glucose, 100 U/mL penicillin, 100 μg/ml streptomycin, and 15% FBS at 37°C in a humidified 5% carbon dioxide atmosphere. The medium was changed every 3 days for CRC cells and twice a week for fibroblasts. Cells were *Mycoplasma* spp.-free.

### Small Extracellular Vesicles Isolation

HCT116 and HT29 cells were plated in T75 flasks (5 × 10^4^ cells/cm^2^) and grown in a complete medium for 48 h until they reached approximately 85–95% confluence. After, cells were washed three times with 0.22-μm filtered PBS and incubated with McCoy’s 5A culture medium without FBS for the following 24 h. After that, the conditioned medium was collected. Conditioned medium was precleared by centrifugation at 4,000 × *g* for 30 min to remove cell debris. The supernatant was filtered through 0.22-μm filtered PBS and concentrated 30× using a Vivaspin Turbo 15 10,000 MWCO (Sartorius, United Kingdom) filter capsule by centrifugation at 4,000 × *g* for 15 min. After that, the concentrates were centrifuged at 15,000 × *g* for 20 min to remove microvesicles (Patel et al., 2019). Next, 0.5 v:v of Total Exosome Reagent (Thermo Fisher Scientific, MA, United States) was added to the concentrated medium and incubated overnight at 4°C under agitation. Next, samples were centrifuged at 10,000 × *g*, 4°C for 1 h. The fraction of pelleted sEVs was washed in 0.22-μm filtered PBS followed by the second step of 10,000 × *g* centrifugation at 4°C for 1 h. Pelleted sEVs were resuspended in 100 μl of 0.22-μm filtered PBS. The remaining cells (EVs donor cells) were scraped and lysed for Western blotting, as described in the following section. We have submitted all relevant data to the EV-TRACK knowledgebase (EV-TRACK ID: EV210162; Van Deun et al., 2017).

### Nanoparticle Tracking Analysis

Estimated concentration and size distribution of nanoparticles in purified isolated sEV samples were determined by nanoparticle tracking analysis using a NanoSight NS300 instrument (Amesbury, United Kingdom) equipped with a 405-nm laser. Each sEV fraction was diluted 1:100 in 0.22-μm filtered PBS to obtain a concentration within the range from 10^7^ to 10^10^ particles/ml. Five different videos of 30 s were recorded for each sample. The temperature was monitored and set at 25°C throughout the measurements. Videos recorded for each sample were analyzed with the NanoSight software version 2.3. For analysis, auto settings were used for blur, screen gain 4, camera level 14, and detection threshold 3.

### Transmission Electron Microscopy

Transmission electron microscopy (TEM) was conducted by the Electron Microscopy Laboratory of the Institute of Biology, University of Campinas. Four microliters of aliquots of resuspended sEVs were applied on 0.5-cm^2^ glow-discharged Formvar/carbon-coated electron microscopy grids and allowed to adsorb at room temperature until a thin-layer film remained. To contrast the samples, grids were transferred to 2% uranyl acetate for 5 min. Grids were left to dry and stored in appropriate grid storage boxes, then observed under an LEO 906 transmission electron microscope (Carl Zeiss, Germany) at 60 kV.

### Lipophilic Labeling of Colorectal Cancer Cell-Derived Small Extracellular Vesicles and Uptake Visualization

Colorectal cancer cell-derived sEVs were fluorescently labeled with PKH26 (Sigma-Aldrich, United States), as described by Torreggiani et al. (2014) with minor modifications. Briefly, sEVs (2 μg resuspended in 10 μl of filtered PBS) or an equal volume of filtered PBS were added to 50 μl of Diluent C. One microliter of PKH26 dye was added to 50 μl of Diluent C before being added to the sEVs and the control. Samples were mixed gently and maintained for 15 min at 37°C before 100 μl of 1% bovine serum albumin (BSA) were added to bind the excess of dye. Samples (PKH26-sEVs and PKH26-PBS control) were then filtered through Exosome Spin Columns (MW3000, Invitrogen, Thermo Fisher, United States) following the manufacturer’s instructions. Human fibroblasts (1 × 10^4^ cells) were cultured in glass coverslips in wells of a 24-well plate. Cells were serum harvested for the next 24 h, and then, PKH26-labeled CRC cell-derived sEVs (2 μg resuspended in 50 μl of DMEM serum-free) or the same volume of PKH26-PBS control were added to the cells for 6, 24, and 48 h. Cells were then washed with PBS and analyzed by fluorescent microscopy. Nuclei were stained with 4′,6-diamidino-2-phenylindole. After 48 h, cells were fixed with 4% paraformaldehyde for 10 min. Actin fibers were stained with ActinGreen 488 (Thermo Fisher, United States) following the manufacturer’s instructions before imaging by Cytation5 (BioTek Instruments).

### Cell Motility

*In vitro* cell migration was performed by classical wound healing assay and transwell migration assay. Wound healing assay was performed following a methodology described by Jana and Trivedi (2018). Human normal fibroblasts (1 × 10^4^ cells) were seeded in a 24-well plate to form a monolayer. Cells were serum harvested for the next 24 h. A scratch was generated using a micropipette tip, and cells were washed with PBS to remove cell debris. Purified CRC cell-derived sEVs (2 μg/ml) in serum-free medium or vehicle (PBS) were added to normal human fibroblasts, and wound healing was monitored by capturing images with Lumascope 620 (Etaluma, Inc) for 24 and 48 h. *In vitro* cell migration assays were performed in a 24-well transwell plate with 8-μm polycarbonate membrane filters (Corning) separating the lower and upper culture chambers. Human fibroblasts were grown to subconfluence. After detachment with trypsin, cells were washed with PBS and resuspended in a serum-free medium, after which the cell suspension (1 × 10^5^ cells) was added to the upper chamber. Purified CRC cell-derived sEVs (2 μg/ml) in serum-free medium or vehicle (PBS) were added to both upper and lower chambers. Cells were maintained in culture conditions for 48 h. The cells that had not migrated were removed from the upper face of the filters using cotton swabs, and the cells that had migrated to the lower face of the filters were fixed with 4% paraformaldehyde for 15 min at room temperature and then permeabilized by 100% methanol for 15 min at room temperature. Cells that had migrated were stained with 0.5% crystal violet solution for 30 min at room temperature. Images of at least five random fields were captured from each membrane using a × 10 objective (Lumascope 620, Etaluma), and the number of migratory cells was counted. All values are representative of at least two independent experiments.

### Signaling Pathway Analysis

Human fibroblasts (5 × 10^5^ cells) were seeded in 100-mm dishes and maintained in culture conditions for 24 h. Next, cells were serum harvested for the following 24 h. Then, cells were co-cultured with purified CRC cell-derived sEVs (2 μg/ml) or equal amounts of PBS for 15 min, 1, 6, 24, and 48 h. Western blotting analysis was performed as described in the next session.

### Western Blotting Analysis

Standard Western blotting was performed according to a published paper (Ferreira et al., 2004) by our group with modifications in the sample preparation steps. Extracellular vesicle (EV)-specific protein markers and non-EV markers in the isolated vesicles and donor cells were examined by Western blotting as also signaling pathway mediators analysis in human fibroblasts co-cultured with CRC cell-derived sEVs. Briefly, CRC cells and human fibroblasts were washed 3× with PBS, scraped, and lysed in a radioimmunoprecipitation assay buffer containing a protease and phosphatase inhibitor for 2 h on ice. Protein extracts were cleared by centrifugation at 14,000 × *g* for 10 min (Hettich Universal 320R, Germany). Pelleted sEVs were directly lysed by radioimmunoprecipitation assay buffer. Protein concentration was determined using the microBCA kit (Thermo Fisher Scientific, MA, United States). A sample buffer (100-mM Tris-hydrochloride pH 6.8, 200-mM β-mercaptoethanol, 4% sodium dodecyl sulfate, 0.1% bromophenol blue, and 20% glycerol) was added to the samples in a 1:1 ratio, and samples were boiled for 10 min. To the sEVs’ samples, a sample buffer under reducing and non-reducing conditions was added (1:1 ratio). For CRC cells, 20 μg of proteins were used; for human fibroblasts, 10 μg of proteins were required, whereas, for EVs characterization, 20 μg of lysed EVs were required. Proteins were applied to sodium dodecyl sulfate–polyacrylamide gel electrophoresis gel (10–12%) and transferred to PVDF membranes. Blocked membranes [3% BSA in Tris–buffered saline (TBS)-Tween 20 (0.05%)] were incubated overnight with specific antibodies at 1:1,000 dilution. After the washing steps in TBS-Tween 20 (0,05%), membranes were incubated with appropriate horseradish peroxidase (HRP)-conjugated secondary antibodies at 1:10,000 dilution in 1% BSA in TBS-Tween 20 (0.05%) for 3 h. Immunoblots were detected by chemiluminescence in Alliance 6.7 (UVITEC, Cambridge, United Kingdom). The following primary antibodies were used: anti-Alix mouse monoclonal (#2171, Cell Signaling); anti-Hrs rabbit monoclonal (#15087, Cell Signaling); anti-Lamp1 rabbit monoclonal (#9091, Cell Signaling); anti-Lamp2 rabbit monoclonal (#49067, Cell Signaling); anti-Lmwptp mouse monoclonal (sc390190, Santa Cruz); anti-Rab11 rabbit monoclonal (#5589, Cell Signaling); anti-Rab27A rabbit monoclonal (#95394, Cell Signaling); anti-Rab27B rabbit polyclonal (GTX64616, GeneTex); anti-Rab5 rabbit monoclonal (#3547, Cell Signaling); anti-Rab7 rabbit monoclonal (#9367, Cell Signaling); anti-Stam2 rabbit monoclonal (#53674, Cell Signaling); anti-Tsg101 rabbit polyclonal (GTX118736, GeneTex); anti-Gm130 rabbit monoclonal (#12480, Cell Signaling); anti-Cd81 mouse monoclonal (GTX43505, GeneTex); anti-α-Tubulin rabbit polyclonal (#2144, Cell Signaling); anti-β-Actin mouse monoclonal (sc47778, Santa Cruz); anti-phospho-Fak (Tyr397; #8556, Cell Signaling); anti-GAPDH (#2118, Cell Signaling); anti-Rock1 rabbit monoclonal (#4035, Cell Signaling); anti-Src rabbit monoclonal (#2123, Cell Signaling); anti-phospho-Src (Tyr527) rabbit polyclonal (#2105, Cell Signaling); anti-RhoA rabbit monoclonal (#2117, Cell Signaling); anti-RhoC rabbit monoclonal (#3430, Cell Signaling); and anti-Rac family rabbit monoclonal (#2465, Cell Signaling). Also, the following secondary antibodies were used: anti-mouse IgG/HRP conjugated (#7076, Cell Signaling) and anti-rabbit IgG/HRP conjugated (#7074, Cell Signaling).

### Fibroblast Activation Assay for α-Smooth Muscle Actin

Activation of normal human fibroblasts with CRC cell-derived sEVs was performed as described by Rai et al. (2019). Briefly, fibroblasts were seeded in glass coverslips (4 × 10^4^ cells) and maintained for 24 h in 400 μl of DMEM high glucose with 15% of EV-depleted FBS (Thermo Fisher, United States). Cells were treated with CRC cell-derived sEVs (10 μg/ml) or vehicle (PBS) for a further 48 h. Cells were then analyzed for α-smooth muscle actin (α-SMA) expression using immunofluorescence assay.

### Immunofluorescence

Immunofluorescence was performed as previously described (Faria et al., 2020) with modifications. Cells were cultured on a glass coverslip on a 24-well plate. Briefly, cells were fixed in 100% methanol for 15 min at 4°C, washed in PBS, and then permeabilized using 0.1% de Triton X-100 in PBS for 15 min at room temperature. Cells were then washed in PBS and blocked with 1% BSA, glycine (22.52 mg/ml) in PBS for 1 h at room temperature. Next, cells were incubated with primary antibody (rabbit anti-α-SMA, 1:100; Cell Signaling; #19245) in 1% BSA and 0.3% Triton X-100 in PBS overnight at 4°C. Coverslips were incubated with secondary antibody (1:500) Alexa Fluor Rabbit 488 (Thermo Fisher Scientific, MA, United States) in 1% BSA and 0.3% Triton X-100 in PBS for 3 h at room temperature in the dark. Nuclei were stained with 4′,6-diamidino-2-phenylindole. Cells were imaged using Lumascope 620 (Etaluma, Inc.).

### Statistical Analysis

Results were expressed as mean ± standard error of the mean. Statistical analysis was assessed using Student’s *t*-test between only two groups. One-way analysis of variance was performed with multiple groups. *P* < 0.05 is considered to be statistically significant. ^∗^*P* < 0.05, ^∗∗^*P* < 0.01, and ^∗∗∗^*P* < 0.001. All data were analyzed using GraphPad Prism Software, version 5.0.

## Results

### Colorectal Cancer Cell Lines With Different Sensitivity to Cytotoxic Agents Display Different Efficiency in Small Extracellular Vesicle Production

**FIGURE 1:**
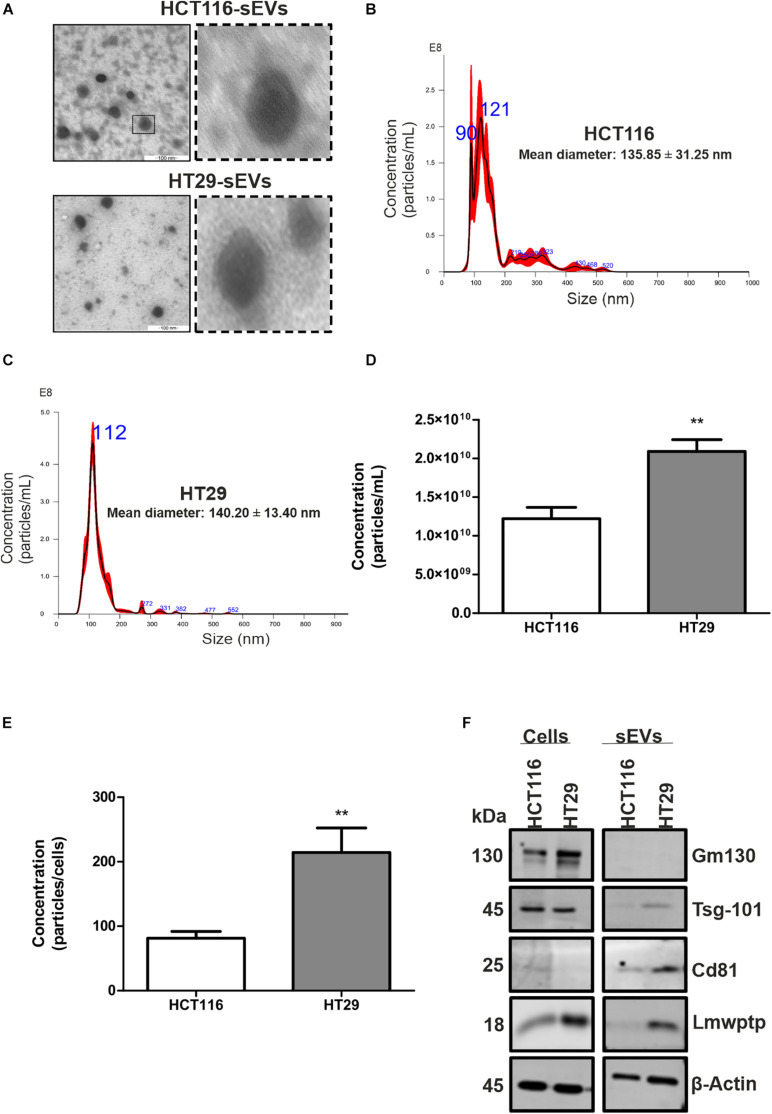
Characterization of sEVs derived from HCT116 and HT29 cells. **(A)** Transmission electron microscopy micrographs of sEVs derived from HCT116 and HT29 cells depicting cup-shaped morphology. Scale bar: 100 nm. Representative graphs illustrating size distribution of sEVs from HCT116 **(B)** and HT29 **(C)** measured by nanoparticle tracking analysis. **(D)** Particle concentration measured by nanoparticle tracking analysis from HCT116- and HT29-derived sEVs. **(E)** Ratio of particle concentration and number of cells. **(F)** Representative images of Western blotting analysis from HCT116 and HT29 whole cell lysates and sEVs isolated from conditioned medium obtained from these cells, showing enrichment of EV markers and absence of non-EVs markers. Lmwptp is a cargo of HT29-derived sEVs (*N* = 3). All values are displayed as mean ± SEM, where **P < 0.01, assessed with Student *t*-test.

We firstly focused on the characterization of EVs for size and purity (Figure 1), which was demonstrated by TEM that these vesicles displayed a compatible and expected morphology for sEVs (Figure 1A). Nanoparticle tracking analysis was used to characterize the size and estimated concentration (number particles/milliliter) of CRC cell-derived EVs. As shown in Figures 1B,C, the isolation protocol applied in this study, which is based on ultrafiltration and polymeric precipitation, purified a heterogeneous population of nanoparticles with mean diameters of 135.850 ± 31.250 and 140.200 ± 13.400, respectively, and a mean mode values of 109.650 ± 7.750 and 104.900 ± 4.200 for HCT116 and HT29, respectively. According to the Minimal Information for Studies of Extracellular Vesicles 2018 (Théry et al., 2019) and to a previous study (Patel et al., 2019) showing a mixture of different vesicle types isolated by this commercial isolation reagent, the use of the term sEVs to describe vesicles smaller than 200 nm was used. A significantly higher concentration of sEVs was released by HT29 as compared with HCT116: 2.090 × 10^10^ ± 1.518 × 10^9^ *vs*. 1.223 × 10^10^ ± 1.453 × 10^9^ particles/ml (*p* = 0.0021; Figure 1D) even if we analyzed the ratio number of particles per cells: 214 ± 38 *vs*. 81 ± 10 (*p* = 0.007; Figure 1E). In addition, we observed that a Western blot analysis was used to identify the presence or absence of a selection of EVs and non-EVs markers to confirm the efficiency of our isolation protocol and the purity of the isolated vesicles. sEVs are characterized by the enrichment of transmembrane proteins associated with the plasma membrane and/or endosomes such as tetraspanins (Cd9, Cd63, and Cd81) and cytosolic proteins recovered in EVs named Alix and Tsg101. Our results showed that CRC cell-derived sEVs were positive for Cd81 and Tsg101 markers and negative for non-EV marker Gm130 (Golgi marker), confirming an enrichment in sEVs in the samples (Figure 1E). We also observed that Lmwptp is a cargo of HT29-sEVs.

### Colorectal Cancer Cell Lines Display Different Levels of Small Extracellular Vesicle Biogenesis Mediators

**FIGURE 2:**
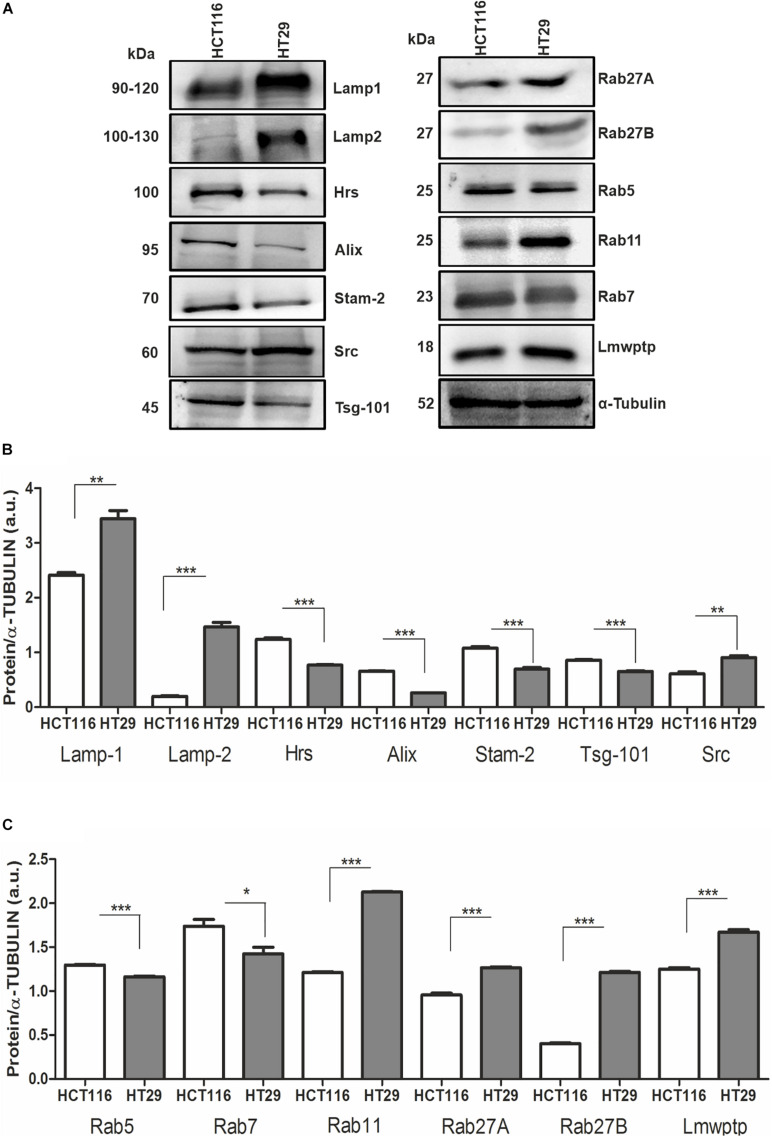
Protein profile from HCT116 and HT29-sEV donor cells. **(A)** Representative images of Western blotting analysis performed for the indicated proteins. α-Tubulin serves as a loading control. **(B,C)** Densitometry values were corrected for loading controls. (*N* = 3 independent experiments). Values are represented as mean ± SEM. *P < 0.05, **P < 0.01, and ***P < 0.001, assessed with Student *t*-test.

Based on the difference in sEV production by CRC cell lines, we then analyzed the protein profile of HCT116 and HT29 at proteins involved in EV biogenesis and transport. HT29 cells displayed a higher amount of lysosomal proteins, Lamp1, Lamp2, and Src protein (Figures 2A,B). It was also observed low intracellular levels of Tsg101, Alix, Hrs, and Stam2 in HT29 compared with HCT116 (Figure 2B). Rab5 and Rab7, components of early and late endosomes, were lower expressed in HT29 compared with HCT116 (Figure 2C). Rab proteins related to EV trafficking, such as Rab11, Rab27A and Rab27B, were significantly enriched in HT29 compared with HCT116 (Figure 2C). Taken together, these expression patterns suggest a differential secretion of EVs subtypes in HT29 cells.

### Small Extracellular Vesicles Released by Colorectal Cancer Cells Are Readily Uptake by Normal Human Fibroblasts

**FIGURE 3:**
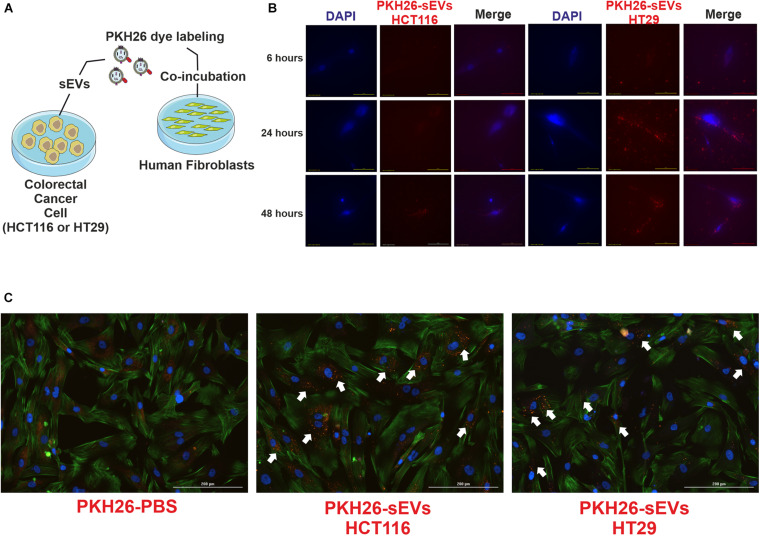
HCT116 and HT29 cell-derived sEVs were uptake by human fibroblasts. **(A)** Schematic diagram of experimental procedures. CRC cell-derived sEVs labeled with PKH26 were added to culture media of human fibroblasts. **(B)** Fluorescent microscopic analysis of normal human fibroblasts incubated with PKH26-stained HCT116-sEVs or HT29-sEVs (2 μg, 6, 24, and 48 h; red). Nuclei were stained with 4′,6-diamidino-2-phenylindole (blue). Scale bar: 100 μm. **(C)** Representative images of human fibroblasts incubated for 48 h with PKH26-stained HCT116-sEVs or HT29-sEVs (2 μg, red); nuclei were stained in blue and actin in green (ActinGreen 488, Thermo Fisher). Scale bar: 100 μm.

Cancer progression is dependent on the crosstalk between tumor cells and the microenvironment, which is enriched in sEVs that are effective on cell-to-cell communication. Then, we investigated the effect of CRC cell-derived sEVs on normal human fibroblasts, expecting to identify a tumor education process mediated by CRC cell-derived sEVs. To demonstrate uptake by fibroblasts (recipient cells), HCT116- and HT29- (donor cells) derived sEVs were labeled with lipophilic tracer PKH26 and incubated with normal human fibroblasts (Figure 3). The tracking of the probe using fluorescence microscopy revealed that both CRC cell-derived sEVs were uptake by normal human fibroblasts within 6 h (Figure 3B), and this uptake remained active until at least 48 h of co-incubation (Figure 3C).

### Colorectal Cancer Cell-Derived Small Extracellular Vesicles Educate Human Fibroblasts and Stimulate Migration

**FIGURE 4:**
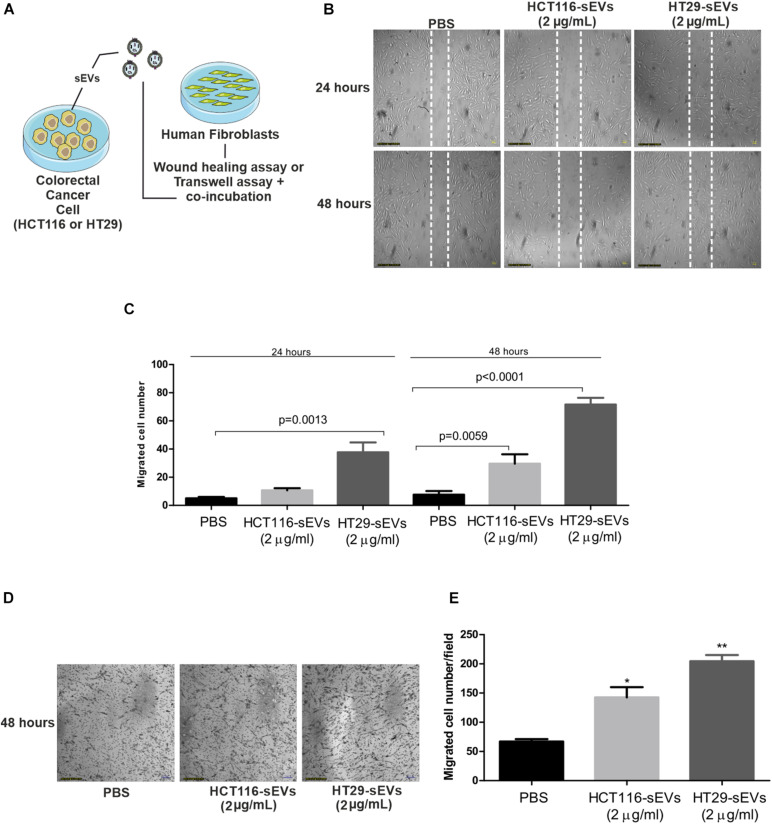
sEVs increase human fibroblast cell migration. **(A)** Schematic diagram of experimental procedures. For wound healing assay, 1 × 10^4^ human fibroblasts were cultured and serum-starved for 24 h. Scratch was made using a pipette tip, and cells were exposed to HCT116 and HT29 cell-derived sEVs (2 μg/ml) or vehicle (PBS) for 24 and 48 h. **(B)** Representative photographs of wound healing assay. **(C)** Quantification of migrated cells. For Transwell assay, human fibroblasts (1 × 10^5^) were incubated for 48 h with HCT116 and HT29 cell-derived sEVs (2 μg/ml) or vehicle (PBS) in serum-free medium. Treatments were incubated both in upper and lower chambers. After 48 h of incubation, migration activity was quantified by counting migrated cells on lower surface of membrane of at least five fields per chamber of a Transwell using a ×10 objective. **(D)** Representative photographs of Transwell migration assay. **(E)** Quantification of migrated cells. All values are representative of mean of at least two independent experiments with similar results and are displayed as mean ± SEM, where *P < 0.05 and **P < 0.01, assessed with Student *t*-test.

The effects of CRC cell-derived sEVs on the migration properties of normal human fibroblasts were confirmed by classical wound healing assay and Transwell system (Figure 4). Scratch assay results showed that both CRC cell-derived sEVs significantly promoted cell migration in 24 and 48 h, which is confirmed by counting the migrated cells to the wound area compared with the PBS group. Interestingly, HT29-derived sEVs promoted the most significant improvement in cell migration (Figures 4B,C). CRC cell-derived sEVs were used as a chemoattractant in a Transwell migration assay to evaluate the ability of normal human fibroblasts to migrate through a pore membrane. Transwell migration assay (Figures 4D,E) indicated that the number of traversed fibroblasts significantly increased with both CRC cell-derived sEVs at 48 h compared with the PBS group, but importantly, it was much more intense when the chemoattractant was HT29-derived sEVs (Figure 4E). It is important to mention that the effect of sEVs in stimulating migration is independent of fibroblast proliferation, observed by BrdU incorporation after 48 h of sEVs co-incubation with human fibroblasts (Supplementary Figure 1).

### Colorectal Cancer Cell-Derived Small Extracellular Vesicles Stimulate Rho-Mediated and Focal Adhesion Signaling

**FIGURE 5:**
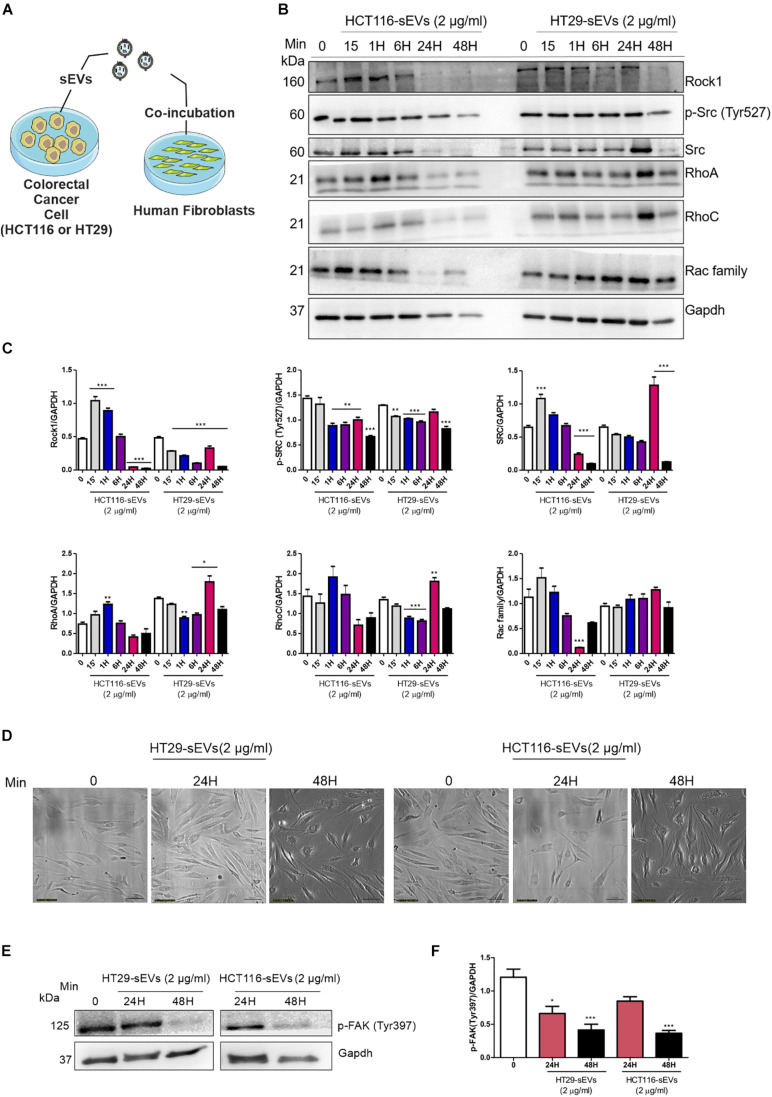
Western Blotting analysis. **(A)** Schematic diagram of the experimental procedures. Human fibroblasts were exposed to one dose of 2 μg/ml of HCT116 and HT29 cell-derived sEVs for 15 min, 1, 6, 24, and 48 h as indicated or vehicle (PBS *t* = 0). **(B,E)** Western blot was used to analyze cell lysates with the indicated antibodies. GAPDH serves as a loading control. **(C,F)** Densitometry values were corrected for loading controls. (*N* = 3 independent experiments). Values are represented as mean ± SEM. *P < 0.05, **P < 0.01, and ***P < 0.001, assessed with one way-ANOVA followed by Tukey post-test. **(D)** Bright field representative images of human fibroblast exposed to HCT116 and HT29 cell-derived sEVs followed each treatment time. Scale bar: 100 μm.

Since it was shown that sEVs from CRC cells were transferred to normal human fibroblasts and stimulated their migration, we examined the relevance of this transference under molecular context, up to 48 h after co-incubation (Figure 5A). Cellular migration is dependent on the strict regulation of assembly and disassembly of focal adhesion sites. The process is mediated by the FAK-Src complex and downstream substrates and regulated by phosphorylation of tyrosine residues of FAK and Src, controlling activation or inhibition. Based on these data, we next examine the underlying mechanism of this enhanced migration, as we can see in Figure 4. It was detected that the amount of Src kinase and its phosphorylated form (Tyr527) dropped in fibroblasts co-incubated with CRC cell-derived sEVs compared with zero point, especially after 48 h of co-incubation by both CRC cell-derived sEVs (Figures 5B,C).

We then analyzed Rho GTPase family proteins. We observed reduced expression of Rho-associated protein kinase (Rock1) in human fibroblasts co-incubated with both CRC cell-derived sEVs (Figures 5B,C), especially after 48 h of co-incubation. RhoA and RhoC presented a similar profile in fibroblasts co-incubated with both CRC cell-derived sEVs. RhoA was elevated at 15 min after co-incubation with HCT116-derived sEVs. Although not reaching statistical significance, we observed a trend toward a reduced expression of RhoA after 24 and 48 h in those fibroblasts co-incubated with HCT116-derived sEVs. RhoC expression did not display significant alterations in those fibroblasts treated with HCT116-derived sEVs. On the other hand, RhoA and RhoC were significantly increased after 24 h of co-incubation with HT29-derived sEVs following a trend toward the reduced expression of RhoA and RhoC in human fibroblasts coculture with HT29-derived sEVs (Figures 5B,C). Rac family was reduced in fibroblasts co-incubated with HCT116-derived sEVs after 24 h, whereas no statistical significance was observed in fibroblasts co-incubated with HT29-derived sEVs.

As seen in Figure 5D, CRC cell-derived sEVs induce morphology changes of human fibroblasts after 24 and 48 h of co-incubation under a light microscope. Our analysis revealed a significant reduction of FAK Tyr397 phosphorylation in human fibroblasts co-incubated with both HCT116- and HT29-derived sEVs in 24 and 48 h compared with the non-treated fibroblasts (Figures 5E,F), thus suggesting an involvement of Rho-FAK signaling.

### Normal Human Fibroblasts Acquire Cancer-Associated Fibroblasts Phenotype by Coculture With Colorectal Cancer Cell-Derived Small Extracellular Vesicles

The morphological changes of fibroblasts triggered by HCT116- and HT29-derived sEVs prompted us to investigate whether these cells were also activated by monitoring α-SMA. Although there are several markers, α-SMA that assembles into contractile filamentous stress fibers running longitudinally, is routinely used as a marker of CAFs.

**FIGURE 6:**
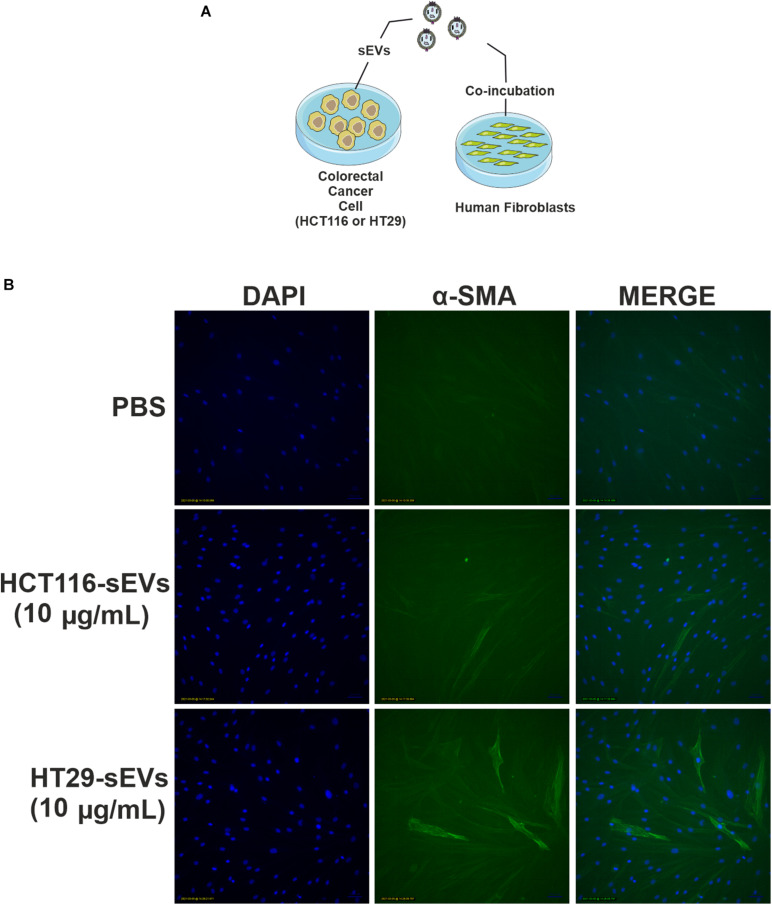
HCT116 and HT29 cell-derived sEVs activate and transform human fibroblasts. **(A)** Schematic diagram of the experimental procedures. **(B)** Fluorescent microscopic analysis of α-SMA (green) expression in human fibroblasts exposed to HCT116 and HT29 cell-derived sEVs (10 μg/ml) or vehicle (PBS) for 48 h. Nuclei were stained with 4′,6-diamidino-2-phenylindole (blue). Scale bar: 100 μm.

To confirm CAF activation, we maintained normal human fibroblasts in culture in the presence of culture medium with EV-depleted FBS, and then, we co-incubated 10 μg/ml of CRC cell-derived sEVs for 48 h (Figure 6A). Fluorescent microscopy revealed that both HCT116-derived sEVs and HT29-derived sEVs induced expression of α-SMA filamentous structures in human fibroblasts (Figure 6B). In contrast, vehicle-treated control human fibroblasts supported only sparse α-SMA filaments. This suggests that CRC cell-derived sEVs, but more robust with HT29-derived sEVs, are capable of triggering activation of normal human fibroblasts.

## Discussion

In recent years, our knowledge regarding sEVs and their role in health and disease have increased drastically. As these vesicles carry a broad spectrum of bioactive molecules, they can communicate with local and distant cells (Valadi et al., 2007; Peinado et al., 2012; Colombo et al., 2014; Rai et al., 2019) and subsequently change the metabolism of these cells (Costa-Silva et al., 2015; Zhao et al., 2016; Zhang et al., 2018). sEVs have emerged as key players in different steps of cancer progression, including invasion, the acquisition of resistant phenotypes, and TME reprogramming (Kahlert and Kalluri, 2013; Chen et al., 2014; Minciacchi et al., 2015; Lugini et al., 2016; Wortzel et al., 2019; Stefanius et al., 2021). Therefore, in the present study, we questioned whether sEVs released by different CRC cell lines could have the same capacity to educate normal human fibroblasts.

Firstly, it was observed that the concentration of EVs produced by HT29 cells was higher compared with HCT116 ones, which was in accordance with molecular markers of biogenesis of these vesicles. It is well known that some tetraspanins, as Cd81 and ESCRT proteins such as Tsg101, are EV-specific markers and are commonly used for EV characterization (Théry et al., 2019). In the present study, the CRC cell-derived EVs were Cd81 and Tsg101 positive with a size smaller than 200 nm. We decided to refer to them as sEVs because the commercial isolation reagent may isolate a mixture of different EVs subtypes (Patel et al., 2019). Furthermore, the vesicle incorporation by normal human fibroblasts cells was confirmed by PKH26 staining, indicating that sEVs could potentially mediate cell-to-cell communication, transferring tumor information from CRC donor cells to normal human fibroblasts (recipient cells).

The literature has shown that tumoral exosomes are able to activate fibroblasts to CAFs. Goulet et al. (2018) studied bladder exosomes that are internalized by normal fibroblasts. The authors observed activation of these fibroblasts revealed by enhanced proliferation and migratory capacity accompanied by a high α-SMA gene expression. In concordance, Zhou et al. (2018) observed that murine fibroblasts have 3T3 internalized PKH26 dye-labeled melanoma-derived exosomes.

In the CRC context, a study conducted by Rai et al. (2019) showed that two distinct CRC cells that release exosomes with differential metastatic capacity could induce different changes in human fibroblasts reprogramming. Both SW480-exos and SW620 induce CAF activation by measuring α-SMA fluorescence. The co-incubation of quiescent fibroblasts with SW480-exos induces high proliferative ability and the secretion of pro-angiogenic factors, whereas the co-incubation of quiescent fibroblasts with SW620-exos induces invasive capacity due to the high expression of MMP11, which are able to remodel the extracellular matrix.

**FIGURE 7:**
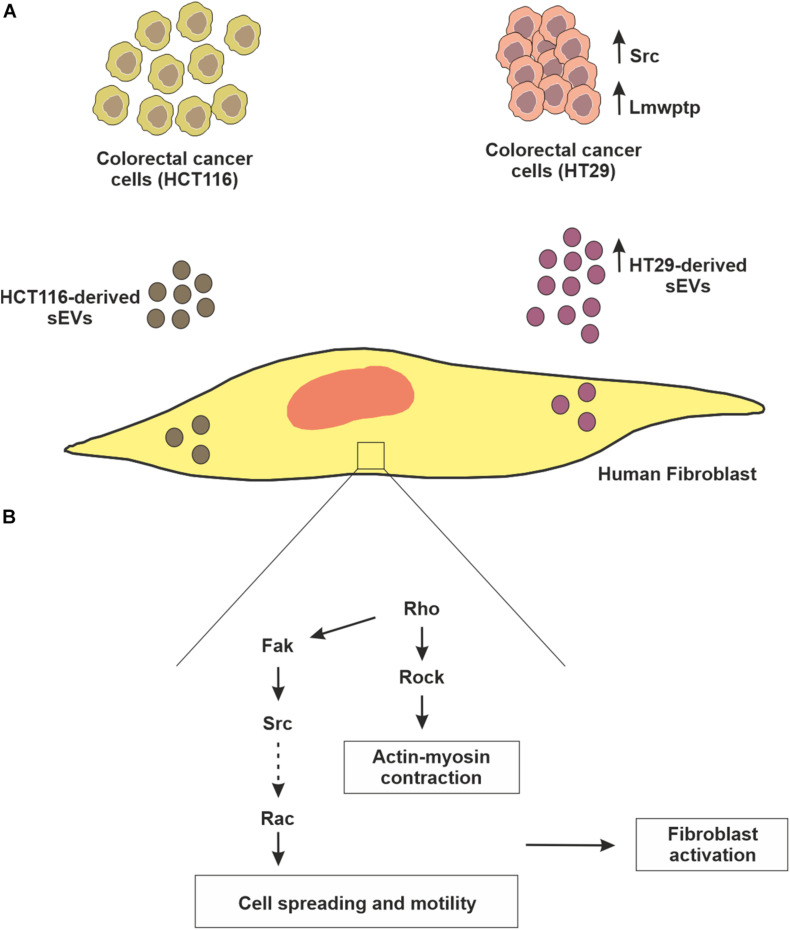
— Schematic representation of fibroblast reprogramming (education) by CRC cell-derived sEVs. **(A)** Although both colorectal cancer cell lines (HCT116 and HT29) release sEVs, HT29 produces these vesicles in greater concentration, which can be explained, at least in part, due to higher amounts of mediators of biogenesis pathway, as well as Src kinase. Interestingly, HT29 not only has a higher amount of Lmwptp, but this protein is part of the sEVs cargo. **(B)** In relation to the biological relevance of these sEVs, in the present study, it was observed that they can educate normal fibroblasts, stimulating them to proliferate, migrate and acquire the characteristic of myoblasts.

We observed an internalization by normal human fibroblasts of PKH26-labeled CRC (HT29 and HCT116 cells)-derived sEVs that culminated in cell migration advantage, which was higher in the case of HT29-derived sEVs. We also have identified that both CRC cell-derived sEVs can activate normal human fibroblasts through the augmented fluorescence of α-SMA in fibroblasts, especially to a greater extent in those co-incubated with HT29-derived sEVs. Our results support the premise that CRC cell-derived sEVs are important players in inducing changes in the fibroblasts’ morphology and inducing migration (Figures 7A,B).

The findings discussed so far demonstrated that the incubation of fibroblasts with sEVs derived from CRC cells (HT29 and HCT116) stimulated three processes: vesicles uptake and fibroblast migration and activation. Increased stromal cells, especially CAFs, are important in extracellular matrix remodeling to enhance tumor growth and the spreading of tumor cells. Actomyosin contractility is a key regulator in actin cytoskeletal reorganization and cell motility (Gunst and Zhang, 2008). To confirm these processes, we analyzed the expression/phosphorylation status of signaling mediators involved in cytoskeleton remodeling. The Rho GTPase network is vital for mediating the intracellular responses that regulate cell adhesion, spreading, and endocytosis (Lawson and Ridley, 2018). Rock controls the formation of stress fibers and focal adhesions in the central part of cultured fibroblasts (Katoh et al., 2001; Katoh, 2017). FAK regulates the organization of new stress fibers and focal adhesions by Katoh (2017) examined the function of FAK at the time of focal adhesion formation in fibroblasts. They observed that Rock activation induced focal adhesion formation in FAK knockout mice. In the same way, Lee et al. (2020) observed a significant reduction of RhoA and Rock1 gene expression in CAFs co-incubated with tumor exosomes isolated from ovarian cancer cells, suggesting that these genes are important in regulating cytoskeletal alterations in CAFs. We observed reduced expression of Rho-associated protein kinase (Rock1) in both human fibroblasts co-incubated with CRC cell-derived sEVs concomitant with reduced phosphorylation of FAK Tyr397. Previous studies demonstrated that the treatment of CAFs, with Rock inhibitor (Y-27632), has smaller focal adhesions (Piltti et al., 2015), which were less stable, resulting in migration (Wang et al., 2017).

Another evidence suggests that p190RhoGEF can also bind to FAK, resulting in increases in RhoA activity contributing to the migration of fibroblasts (Lim et al., 2008). Our results showed reduced expression of RhoA and RhoC at 48 h, followed by co-incubation of CRC cell-derived sEVs with human fibroblasts, especially with HT29-derived sEVs. Chiarugi et al. (2000) used a model of Lmwptp overexpression in 3T3 fibroblasts, which results in downregulation of p190Rho-GAP, a Rho signaling downstream protein related to cytoskeleton rearrangement. In agreement with FAK’s role in fibroblasts migration, a study conducted by Rigacci et al. (2002) showed reduced focal adhesion and high motility capacity in Lmwptp overexpressed fibroblasts due to a reduced p125FAK phosphorylation.

Focal adhesions are integrin-based adhesions linking the actin cytoskeleton to the extracellular matrix, allowing the cell to sense and adapt to abrupt changes in their environment (Geiger et al., 2009). Tyrosine phosphorylation is a key signaling event at the assembly and disassembly of focal adhesions. Tyrosine phosphorylation of FAK recruits several different adhesion proteins by providing docking sites for SH2-containing proteins, such as the Src family (Xing et al., 1994). Downstream signaling can alter cell cytoskeletal dynamics to produce mechanical force for cell motility, and these pathways are under the control of tyrosine kinases and tyrosine phosphatases. As we observed that the FAK Tyr397 phosphorylation decreased after 24 and 48 h of co-incubation of human fibroblasts with CRC cell-derived sEVs and this phospho-residue creates new sites to Src interaction, we next checked Src kinase. Interestingly, a reduced Src expression, as well as its phosphorylation at Tyr527 (inhibitory site), was detected at 48 h after CRC cell-derived sEV co-incubation with human fibroblasts. This reduced phosphorylation of Src Tyr572 might culminate in Src activation, but the reduced phosphorylation of FAK Tyr397 might not create new sites for Src interaction, despite its activity. The literature mentioned that PTPN12, a kind of phosphatase, dephosphorylates FAK, thus reducing the number of focal contacts and consequently increasing tumor cell migration and invasion (Zheng et al., 2009, 2011).

Our group has shown that the Lmwptp contributes to CRC cells’ aggressiveness, low response to chemotherapy, and migration. In addition, this enzyme expression follows a stepwise augment from healthy tissue to dysplastic adenoma and carcinoma, and therefore, we claimed that it could be understood as a biomarker of aggressiveness and poor prognosis for CRC patients (Hoekstra et al., 2015). In this study, we show for the first time that a CRC cell line (HT29) that is less sensitive to therapeutics (Lopes-Costa et al., 2017; Attoub et al., 2018) not only produces more sEVs (Figure 7) but also releases the Lmwptp as a cargo.

## Conclusion

An emerging physiological significance of tumoral sEVs is to transfer a “prototype” message that reflects mutations, genetics, and protein profile from donor cells to recipient cells in the TME. The present study revealed that sEVs produced by CRC cells not only are able to educate normal fibroblasts *via* uptake of these vesicles but also modulate Rho and FAK, which culminate in migration and activation of fibroblasts (Figure 7). Of great interest, the Lmwptp was found to be part of cargoes of CRC cell-derived sEVs. This observation opens new avenues regarding the molecular mechanism by which this phosphatase gives an advantage to CRC.

## Data Availability Statement

The raw data supporting the conclusions of this article will be made available by the authors, without undue reservation.

## Author Contributions

SPC designed the methodology and conducted the research investigation. SRC designed and conducted the TEM methodology. The head group leader CVF-H contributed to the article conceptualization. SPC was responsible for the original writing. SPC and CVF-H were responsible for reviewing and editing. CVF-H, MP, and GF supervised the work. All authors read and approved the final manuscript.

## Conflict of Interest

The authors declare that the research was conducted in the absence of any commercial or financial relationships that could be construed as a potential conflict of interest.
