# Supplementary material for: Colorectal Cancer Cell-Derived Small Extracellular Vesicles Educate Human Fibroblasts to Stimulate Migratory Capacity
Source: Front Cell Dev Biol. 2021 Jul 15;9:696373. doi: 10.3389/fcell.2021.696373 (PMC8320664; doi:10.3389/fcell.2021.696373)
Supplement: Supplementary file 1 [file Data_Sheet_1.PDF]

Supplementary Methods

BrdU measurement

BrdU assay was conducted according to the standard protocol of manufacturer - CytoSelect BrdU cell proliferation ELISA kit (CellBiolabs). Briefly, $2.5x10^3$ human normal fibroblasts were seeded in a 96-well plate and cultured for 24 hours. Cells were serum harvested for the next 24 hours. Next, purified CRC cell-derived sEVs (2 µg/ml) in serum-free medium or vehicle (PBS) were added to human normal fibroblasts and incubated for the following 48 hours. BrdU 10x was added to the wells and incubated for 2 hours at 37°C. The cells were then fixed for 30 minutes at 37°C, followed by washing with specific buffer and incubated with anti-BrdU antibody (1:1000) for 1 hour at room temperature. After washing, cells were incubated with Secondary Antibody HRP Conjugate (1:1000) at room temperature for 1 hour on an orbital shaker. The wells were rinsed three times with the washing buffer and the substrate solution was added. The stop solution was added, and the absorbance was read at 450 nm.

Supplementary Figure

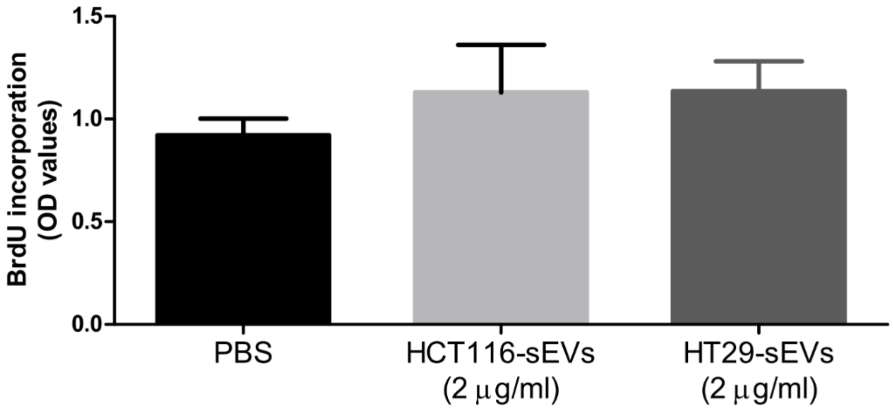

Supplementary Figure 1. Human fibroblast proliferation measured by BrdU incorporation after co-incubation with HCT116 and HT29 cell-derived sEVs (2 μg/ml) or vehicle (PBS) for 48 hours. The absorbance was read at 450 nm.
